# Medical News

**Published:** 1855-03

**Authors:** 


### THE MEDICAL EXAMINER.

PHILADELPHIA, MARCH, 1855.

### MEDICAL NEWS.

We regret to observe that Drs. D. J. Cain and F. Peyre Porcher have retired from the editorship of the *Charleston Medical Journal and Review.* Their loss will be widely felt by the profession. Dr. C. Happoldt, who has afforded material aid to the Journal for the last eighteen months, and who is otherwise favorably known to its readers, has assumed the management of the Journal. We heartily wish him success in his undertaking.

Dr. George Stevens Jones has resigned his editorial connection with *The Boston Medical and Surgical Journal.* The Editorial duties of the Journal will hereafter be assumed by Drs. Wm. W. Morland and Francis Minot. These gentlemen are well known to the profession and we are confident that the Journal will present even increased attractions in their hands.

Dr. Bennet Dowler, whose charge of *The New Orleans Medical and Surgical Journal* has just expired, proposes to issue a Quarterly Journal in May next, to be entitled The New Orleans Quarterly Journal of Medicine. Each number will contain two hundred and sixteen pages, octavo. We earnestly hope that this project, for which Dr. Dowler's industry and abilities so well fit him, will prove a successful one.

To an obituary notice, extracted from the *London Lancet*, of Dr. Golding Bird, by one of our cotemporaries, the following editorial comments are attached : "To which we may add that Dr. G. Bird was the author of works of a lighter nature, as "Nick of the Woods," and other novels which are well known." Our esteemed cotemporary is no ornithologist, or he would be aware that there are many kinds of Birds. The one he alludes to, the author of "Calavar," &c., was a native of Philadelphia.

In the Record Department of the January number of our respected cotemporary *The Western Journal of Medicine and Surgery*, Dr. Mason's paper, entitled "Case of Rupture of the Uterus, &c.," is incorrectly accredited to the operator, Dr. John Neill. The usual crêdit, also, is not given; both of which oversights we have no doubt will be corrected.

---

Dr. Moreton Stillé has been elected to the post of Lecturer on the "Theory and Practice of Medicine," in the Philadelphia Association for Medical Instruction, a position ably and satisfactorily occupied for many years by Dr. J. Forsyth Meigs. We congratulate the Association upon the excellent choice they have made.

---

The following gentlemen have been duly appointed to represent the College of Physicians at the meeting of the American Medical Association: Drs. G. B. Wood, J. Rodman Paul, Isaac Hays, John Neill, F. G. Smith, Bernard Henry, E. Hartshorne, G. W. Norris, Franklin Bache, R. A. Given, Francis West, P. B. Goddard.

---

The St. Joseph's Hospital, of this city, has elected Dr. Alfred Stillé as its delegate to the American Medical Association.

---

*Dr. Séquard.*—The numerous friends of this gentleman will be pleased to see, from the accompanying letter, that he has arrived in this country, and assumed the physiological chair in the Medical College of Virginia. His continued investigations, with regard to the functions of the pneumo-gastric nerves, have afforded results which are now, for the first time presented to the public:

RICHMOND, January 21st, 1855.

DEAR DOCTOR:—Will you have the kindness to give room to the following lines in the next number of your Journal?

I wish to *take date* of the discovery of two important facts relative to the physiology of the pneumo-gastric nerves.

I had already found and published that these nerves are the motor nerves of the vessels of the heart; I have since found that they are also the principal (if not the only) motor nerves of the small vessels of the ungs. This view is founded on the following facts:

1st. The section of the pneumo-gastric nerves, in the cervical region, is followed by the dilatation (or paralysis) of the small vessels of the lungs, as well as it is by the dilatation of the vessels of the heart.

2d. The galvanization of these nerves in the cervical region, after they have been carefully separated from the sympathetic, produces the contraction of the vessels of the lungs, as well as that of the vessels o the heart.

I will prove elsewhere that the dilatation of both the vessels of the lungs and those of the heart, which follows the section of the pueumogastric nerves, is the first and principal cause of the death, which is the constant result of this operation. Yours, very cordially,

E. Brown Séquard, M. D.,

Dr. McCaw *Prof. Inst. Med. etc. in the Med. Col. of Va.*

Virginia Med. and Surg. Journal.

